# Hypermethylation and decreased expression of *TMEM240* are potential early-onset biomarkers for colorectal cancer detection, poor prognosis, and early recurrence prediction

**DOI:** 10.1186/s13148-020-00855-z

**Published:** 2020-05-12

**Authors:** Shih-Ching Chang, Phui-Ly Liew, Muhamad Ansar, Shih-Yun Lin, Sheng-Chao Wang, Chin-Sheng Hung, Jian-Yu Chen, Shikha Jain, Ruo-Kai Lin

**Affiliations:** 1 Division of Colon and Rectal Surgery, Department of Surgery, Taipei Veterans General Hospital, Taipei, Taiwan, Republic of China; 2 Department of Pathology, Shuang Ho Hospital, Taipei Medical University, New Taipei, Taiwan, Republic of China; 3 Department of Pathology, School of Medicine, College of Medicine, Taipei Medical University, Taipei, Taiwan, Republic of China; 4 Ph.D. Program for the Clinical Drug Discovery from Botanical Herbs, Taipei Medical University, Taipei, Taiwan, Republic of China; 5 Graduate Institute of Pharmacognosy, Taipei Medical University, Taipei, Taiwan, Republic of China; 6 Ph.D Program in Biotechnology Research and Development, College of Pharmacy, Taipei Medical University, Taipei, Taiwan, Republic of China; 7 Department of Surgery, School of Medicine, College of Medicine, Taipei Medical University, Taipei, Taiwan, Republic of China; 8 Division of General Surgery, Department of Surgery, Shuang Ho Hospital, Taipei Medical University, New Taipei City, Taiwan, Republic of China; 9 School of Pharmacy, College of Pharmacy, Taipei Medical University, Taipei, Taiwan, Republic of China; 10 Master Program for Clinical Pharmacogenomics and Pharmacoproteomics, Taipei, Taiwan, Republic of China; 11 Clinical trial center, Taipei Medical University Hospital, Taipei, Taiwan, Republic of China

**Keywords:** *TMEM240*, DNA methylation, Early onset, Prognostic marker, Circulating cell-free DNA, ccfDNA, cmDNA, Early detection

## Abstract

**Background:**

Gene silencing by aberrant DNA methylation of promoter regions remains the most dominant phenomenon occurring during tumorigenesis. Improving the early diagnosis, prognosis, and recurrence prediction of colorectal cancer using noninvasive aberrant DNA methylation biomarkers has encouraging potential. The aim of this study is to characterize the DNA methylation of the promoter region of *TMEM240*, as well as gene expression and its effect on cell biological functions and its applications in early detection and outcome prediction.

**Results:**

Highly methylated CpG sites were identified in the *TMEM240* gene by Illumina methylation 450K arrays in 26 Taiwanese patient paired samples and 38 paired samples from The Cancer Genome Atlas (TCGA) colorectal cancer dataset. Transient transfection and knockdown of *TMEM240* were performed to demonstrate the role of TMEM240 in colorectal cancer cells. The data showed that TMEM240 could lead to G1 cell cycle arrest, repress cancer cell proliferation, and inhibit cancer cell migration. The quantitative methylation-specific real-time polymerase chain reaction (PCR) results revealed that 87.8% (480 of 547) of the colorectal cancer tumors had hypermethylated *TMEM240*, and this was also found in benign tubular adenomas (55.6%). Circulating cell-free methylated *TMEM240* was detected in 13 of 25 (52.0%) Taiwanese colorectal cancer patients but in fewer (28.6%) healthy controls. In 72.0% (85/118) of tissue samples, *TMEM240* mRNA expression was lower in Taiwanese CRC tumor tissues than in normal colorectal tissues according to real-time reverse transcription PCR results, and this was also found in benign tubular adenomas (44.4%). The TMEM240 protein was analyzed in South Korean and Chinese CRC patient samples using immunohistochemistry. The results exhibited low protein expression in 91.7% (100/109) of tumors and 75.0% (24/32) of metastatic tumors but exhibited high expression in 75.0% (6/8) of normal colon tissues. Multivariate Cox proportional hazards regression analysis found that mRNA expression of *TMEM240* was significantly associated with overall, cancer-specific, and recurrence-free survival (*p* = 0.012, 0.007, and 0.022, respectively).

**Conclusions:**

Alterations in *TMEM240* are commonly found in Western and Asian populations and can potentially be used for early prediction and as poor prognosis and early-recurrence biomarkers in colorectal cancer.

## Introduction

Colorectal cancer (CRC) is the most commonly diagnosed cancer in the USA and Taiwan [1, 2]. In the USA and Taiwan, CRC has been the most common cancer for the past 9 years and is now also the third leading cause of cancer deaths in females and males [1–3]. CRC is curable in ~ 90% of cases if it is detected at an early stage. The early detection of CRC through screening programs that detect mucosal changes that are predictive of colorectal tumors reduces the incidence and mortality rates of this disease [4]. CRC is a multifactorial disease that arises from an accumulation of acquired genetic and epigenetic alterations in a number of oncogenes, tumor-suppressor genes (TSGs), mismatch-repair genes, and cell cycle genes during tumorigenesis [5–8]. Among the epigenetic alterations, gene silencing by aberrant DNA methylation of promoter regions remains the most dominant phenomenon occurring during tumorigenesis [9]. Regardless of the biological consequences of methylation-induced silencing of TSGs, this epigenetic alteration constitutes a molecular signature that can serve as a promising biomarker for early detection [8, 10]. Circulating cell-free DNA (ccfDNA) levels reveal a greater dynamic range and have a better correlation with changes in tumor burden than CA-153 or circulating tumor cells [11]. ccfDNA methylation assessment allows for simultaneous screening of CRC complementing current modalities, increasing compliance, and cost-effectiveness [12]. *Septin 9* (*SEPT9*) hypermethylation was previously reported to be used for the early prediction of CRC [13]. *SEPT9* methylation in circulating cell-free (ccfDNA) was approved by the US Food and Drug Administration (FDA) as a biomarker for CRC early diagnostic screening. The sensitivity for stages I~III CRC is 64% (48%~77%) [14]. In a Taiwanese CRC cohort, a quantitative polymerase chain reaction (qPCR) revealed that 86.1% of CRC patients exhibited hypermethylated *BEND5* [15], suggesting that more sensitive biomarkers were worth investigating in Asian cohorts. In addition, assays of circulating methylated DNA (cmDNA) could be used as outcome predictors in chemotherapy and multikinase inhibitor-treated metastatic CRC patients [16]. These findings encouraged us to identify additional potential CRC-specific methylation markers, and combining multiple biomarkers in the early stage of CRC screening may provide more sensitive CRC early diagnoses [6, 9]. In our investigation, the human Methylation 450K array was used in 26 paired Taiwanese CRC tissues to identify a new potential CRC-specific hypermethylated transmembrane-encoding gene *TMEM240*.

The *TMEM240* gene encodes a transmembrane domain-containing protein found in the brain and cerebellum. Mutations of *TMEM240* were found to cause spinocerebellar ataxia 21 (SCA21) with mental retardation, severe cognitive impairment, and hypokinetic and hyperkinetic movement disorders in patients from France, Germany, Holland, Colombia, Japan, and China [17–20]. The mechanism of the pathogenesis of SCA21 may be mediated through the induction of early gliosis and lysosomal impairment by mutant *TMEM240* [21]. Genome-scale analysis of DNA methylation using Infinium HumanMethylation450 BeadChips found hypermethylation of *TMEM240* in 22 CRC patients in Russia [22]. However, the role of TMEM240 in tumorigenesis is unclear. Few studies have analyzed the overall methylation status and expression of *TMEM240* or its biological functions, clinical significance, or application. Therefore, this study investigated the methylation, expression level, biological functions, clinical significance, and clinical applications of *TMEM240* in CRC.

## Results

### Four potential candidate genes were identified from Taiwanese and Western CRC patients by genome-wide methylation analysis

**Fig. 1:**
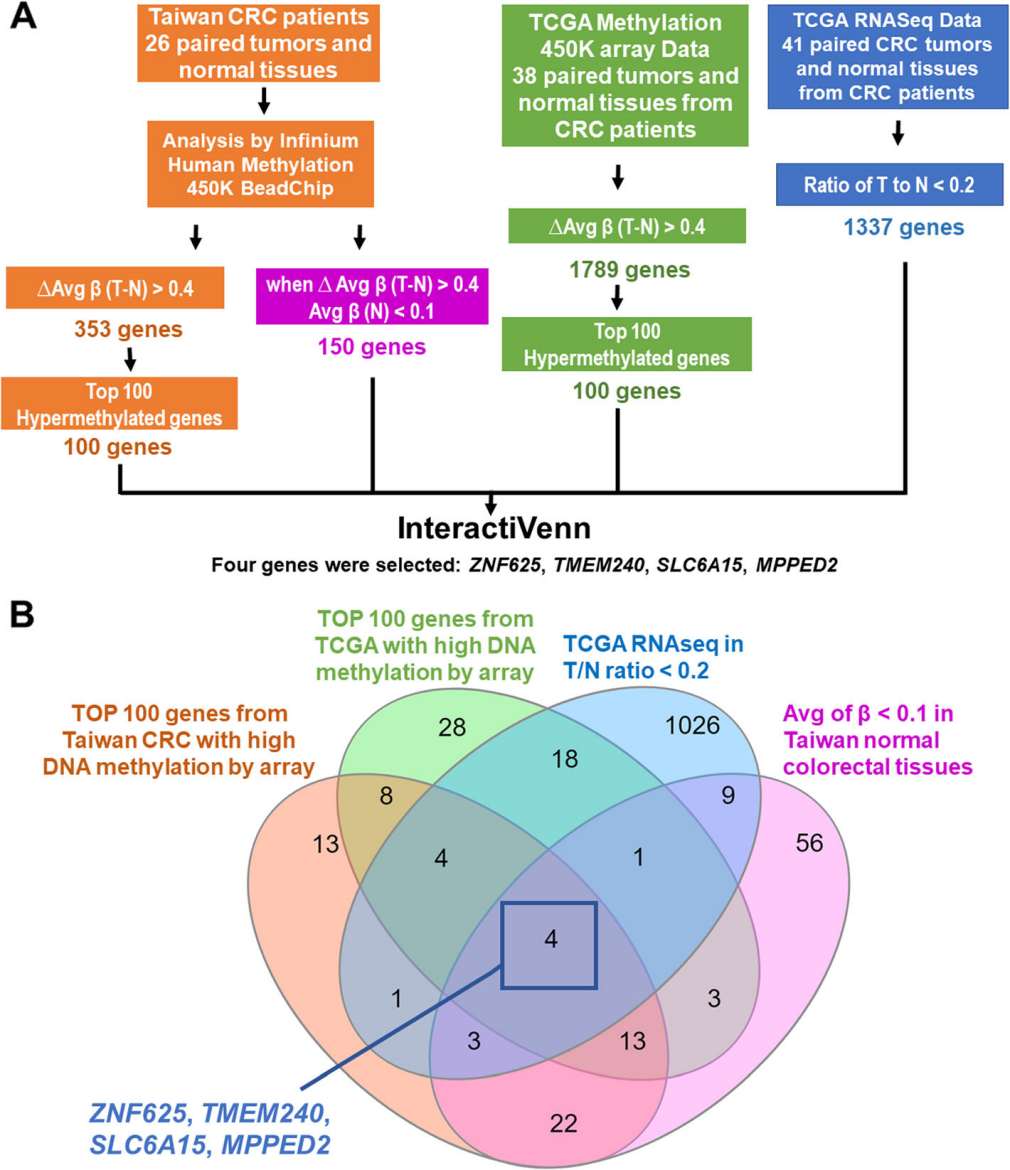
Flowchart of gene selection and analytical procedures. **a** The criteria and step-by-step flowchart for gene selection. **b** Screening of intersecting genes by InteractiVenn

To identify novel potential biomarkers in CRC patients, we used four criteria to screen potential targets: (1) hypermethylation in Taiwanese CRC patients, (2) a methylation level in normal colorectal tissues of close to 0, (3) hypermethylation in Western CRC patients, and (4) low expression in CRC (Fig. 1a). First, the Illumina Infinium HumanMethylation450 BeadChip array was applied to identify critical TSGs from 26 CRC tissues and paired noncancerous colorectal tissues. In total, 353 genes were found to be hypermethylated when the ΔAvg _β (βTumor–βNormal) was > 0.4. Second, among the 353 genes, 150 genes were selected when the Avg _β value was very low in noncancerous colorectal tissues (Avg _β (Normal) was < 0.1). Next, we analyzed the TCGA Illumina Infinium HumanMethylation450 BeadChip array data of 38 paired Western CRC patients. In total, 1789 genes were found to be hypermethylated when the ΔAvg _β (βTumor–βNormal) was > 0.4. Fourth, the TCGA RNA sequencing data of 41 paired Western CRC patients revealed that the expression levels of 1337 genes were decreased by 80% in CRC. Four candidate genes, *ZNF625*, *TMEM240*, *SLC6A15*, and *MPPED2*, were identified using InteractiVenn (Fig. 1b). ZNF625, SLC6A15, and MPPED2 have also been found to be hypermethylated in the tumors of CRC patients in previous studies [23–25]. MPPED2 was reported to work as a tumor suppressor gene [24, 26, 27]. Few reports about TMEM240 in cancer were found. Analysis of the methylation status in most human digestive systems showed that *TMEM240* was frequently hypermethylation in esophageal cancer (12/15, 80.0%), gastric cancer (2/2, 100%), colon cancer (38/38, 100%), rectal cancer (5/5, 100%), liver cancer (37/46, 80.4%), and pancreatic cancer (4/9, 44.4%), compared to paired adjacent normal tissues (Figure S1). Thus, the role of TMEM240 in cancer was unclear. Therefore, TMEM240 in CRC was selected for further analysis. Comprehensive analyses of epigenetic alterations, mRNA and protein expression, and the biological role of TMEM240 were further performed.

### TMEM240 protein represses colon cancer cell proliferation and migration

**Fig. 2:**
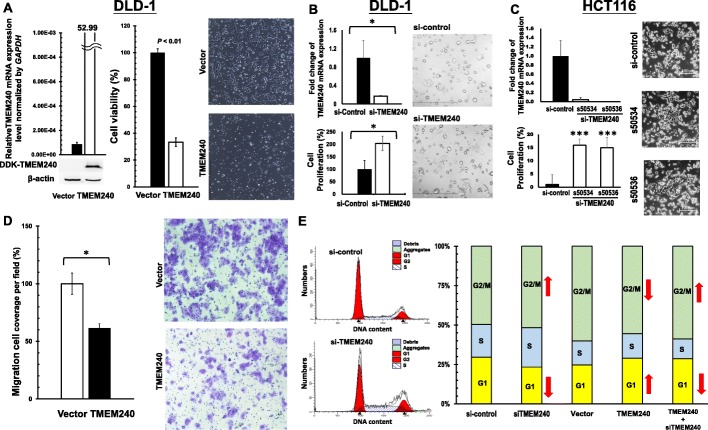
TMEM240 may repress cell growth, migration, and cell cycle arrest in colon cancer cells. **a** A recombinant pMyc-DDK-hTMEM240 plasmid was transfected into DLD-1 cells for 24 h, and then the cells were analyzed via real-time RT-PCR for mRNA and Western blotting for protein (left). The cell proliferation rate was analyzed by sulforhodamine B (SRB) assay (middle). A bright view was taken to illustrate the cell morphology (right) (original magnification, × 100). **b** si-TMEM240 was transfected into DLD-1 cells. The mRNA expression (up), cell proliferation rate (down), and cell morphology (right) of DLD-1 cells were analyzed (original magnification, × 100). **c** The mRNA expression (up), cell morphology (right), and cell proliferation rate (down) in HCT116 colon cancer cells were analyzed (original magnification, × 40). **d** The migratory ability was measured by a transwell assay after *TMEM240* overexpression in DLD-1 cells. **e** A *TMEM240* and/or si-TMEM240 plasmid was transfected into DLD-1 cells for 24 h, and then the cell cycle distribution was analyzed by flow cytometry. Data are presented as the mean ± SD, *p ≤ 0.05, **p ≤ 0.01, ***p ≤ 0.001. A *t* test was used to calculate group differences in all experiments. Experiments were performed with at least two biological duplicates and three technical replicates

Alterations in TMEM240 and its functional roles during tumorigenesis had not been previously studied. To study the biological roles of the TMEM240 protein in CRC cells, a TMEM240 plasmid or si-TMEM240 was transfected by the electroporation systems into DLD-1 cells which exhibited low level of TMEM240 expression (Figure S2). The gene manipulation efficiency was determined through real-time RT-PCR and Western blotting. Transfection of the *TMEM240* plasmid into the DLD-1 cell line resulted in an abundant display of *TMEM240* mRNA and protein by DLD-1 cells. According to the cell viability sulforhodamine B (SRB) assay, the growth of DLD-1 cancer cells with TMEM240 was slow down by 66.6% compared to that of the vector control group (Fig. 2a). Microscopic observations revealed that TMEM240 overexpression repressed the growth of DLD-1 cells. To verify whether the decrease in TMEM240 expression induced cell growth, knockdown of *TMEM240* gene expression was achieved in the DLD-1 cell line with si-TMEM240. *TMEM240* knockdown increased DLD-1 cell growth by 2.0-fold (Fig. 2b). We further transfected with two si-TMEM240 (s50536) and si-TMEM240 (s50534) into the colon cancer cell line HCT116, respectively. *TMEM240* mRNA expression was dramatically decreased by two si-TMEM240, s50534 and s50536. The microscopy and SRB assays showed that both si-TMEM240 s50534 and s50536 could significantly induce colon cancer growth by 15.9- and 14.9-fold, respectively (Fig. 2c).

To investigate whether TMEM240 was associated with colon cancer cell migration, *TMEM240* was overexpressed in DLD-1 cells for 24 h. Next, cell motility was analyzed using transwell assays. The data revealed that the increase in TMEM240 suppressed the migratory ability of DLD-1 cells by 39.7% (Fig. 2d).

### TMEM240 expression induced cancer cell arrest in the G_1_ phase

The data of Fig. 2 have indicated that the *TMEM240* gene inhibits the growth of cancer cells and that the growth of cells requires regulation of the cell cycle. Therefore, cell cycle regulation by TMEM240 was studied by flow cytometry. The percentage of DLD-1 cells in the G_1_ phase increased by 4.28% in *TMEM240*-knockdown cells compared to the vector control. *TMEM240* knockdown induced a decrease in the proportion of cells in the G_1_ phase by 6.33% compared to the siRNA control. An opposite trend was observed for the G_2_M phase, i.e., the percentage of DLD-1 cells in the G_2_M phase decreased by 4.53%, and *TMEM240*-knockdown induced a 2.05% increase in the proportion of cells in the G_2_M phase compared to the control (Fig. 2e).

### Hypermethylation of *TMEM240* in CRC tumors and plasma from Taiwanese CRC patients

**Fig. 3:**
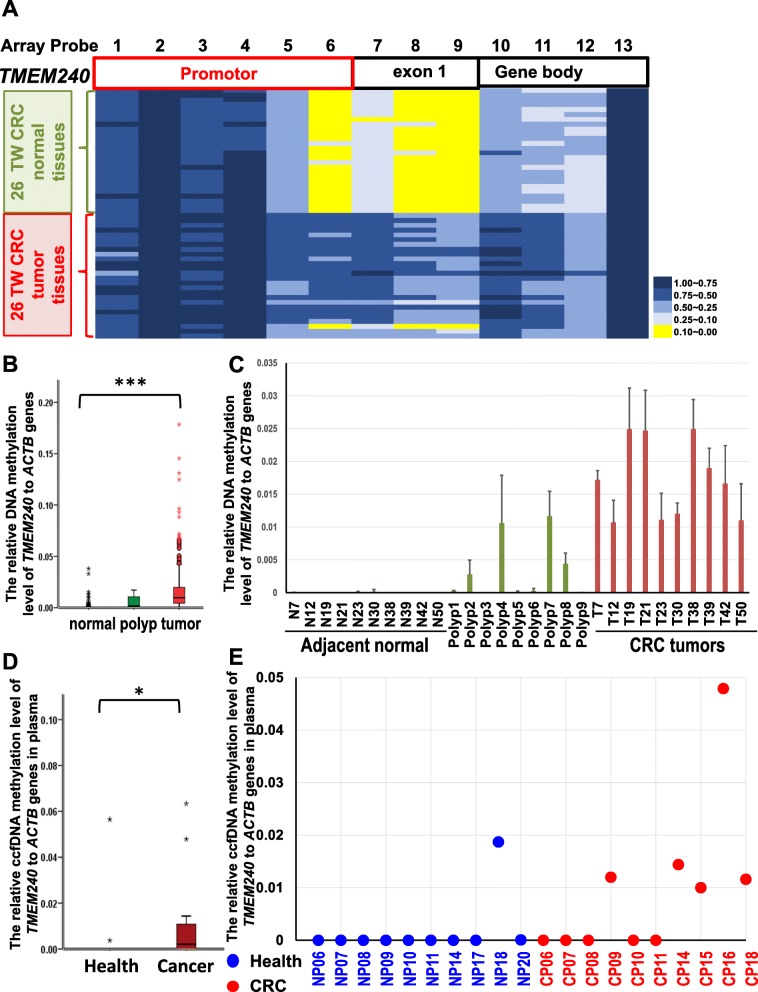
Methylation levels in Taiwanese colorectal carcinoma (CRC) patients. **a** Differentially methylated CpG heatmap of *TMEM240* in 26 paired CRC patients. Methylation levels (average β values) at differentially methylated loci were identified using an Illumina Methylation 450K array-based assay. The highly differentially methylated sites cg15487867, cg16601494, cg16306898, and cg22497741 are array probes 6, 7, 8, and 9, respectively. The genomic positions of the QMSP amplicon are located in exon 1 (from + 171 to + 347) of the *TMEM240* gene (array probes 7 and 8). **b** The box plot of *TMEM240* methylation levels in tissues. **c** Representative figures of *TMEM240* methylation levels determined by QMSP in 10 adjacent normal colon tissues, nine polyps of tubular adenoma, and 10 CRC tumors. Experiments were performed with three technical replicates. **d** The box plot of *TMEM240* methylation levels in plasma. **e** Representative figures of the circulating methylated *TMEM240* levels determined by QMSP in 10 healthy subjects and 10 CRC patients. *p ≤ 0.05, ***p ≤ 0.001. A *t* test was used to calculate group differences

TMEM240 was found to have tumor suppressor potential via affecting CRC cell growth and migration (Fig. 2) and was found to be hypermethylated in both Taiwanese and Western CRC patients (Fig. 1). Therefore, we further comprehensively analyzed DNA methylation alterations and mRNA and protein expression in Asian and Western CRC patients. In 26 paired Taiwanese CRC patients, the Illumina Infinium HumanMethylation450 BeadChip array found three methylation differences in the ΔAvg _β (βTumor–βNormal) between paired CRC tumor and adjacent normal tissues at of the cg16601494, cg15487867, and cg16306898 sites, and the ΔAvg _β values at these sites were 0.47, 0.45, and 0.41, respectively, in the promoter and exon regions of the *TMEM240* gene. In the *TMEM240* gene, 30 CpG sites were identified, and an increase in the methylation level was found mainly in the promoter and exon 1 regions, most significantly in CpG sites, in CRC tumors compared to adjacent normal tissues, as shown in a heatmap in Fig. 3a.

Methylation patterns of *TMEM240* were verified by performing QMSP assays in 547 CRC patients and nine patients with benign tubular adenoma. The median DNA methylation levels that were normalized by *ACTB* were 0.0098 in CRC tumors, 0.0015 in benign tumors, and 0.0001 in adjacent normal tissues (Fig. 3b, Figure S3, Table 1). The data indicated that in 87.8% (480/547) of Taiwanese CRC patients, the methylation level of *TMEM240* was at least 5-fold higher in tumor tissues than in the matched normal colorectal tissues (Fig. 3 b and c, Table 1). Hypermethylation of the *TMEM240* promoter was detected in five of nine (55.6%) patients with a benign tubular adenoma (Table 1). DNA hypermethylation of *TMEM240* is especially higher in the cecum or appendix and transverse colon than in other locations (Table 1). In addition, in plasma, the median DNA methylation levels were 0.0021 in CRC patients and 0.0000 in healthy controls. The hypermethylation of the *TMEM240* promoter was detected in plasma circulating cell-free DNA from 13 of 25 (52.0%) CRC patients, but less *TMEM240* promoter hypermethylation was detected in healthy subjects (12 of 42, 28.6%, Fig. 3 d and f).

**Table 1:** Alterations of *TMEM240* in relation to the clinical parameters of Taiwan CRC cancer

Characteristics	Total ***n***^**a**^	***TMEM240*** methylation^**b**^				Total ***n***	***TMEM240*** mRNA^**c**^				Total ***n***	TMEM240 protein			
							Low ***n*** (%), high ***n***(%)					Low ***n*** (%), high ***n*** (%)			
		Low ***n*** (%), high ***n*** (%)													
**Overall**	556	71	(12.8)	485	(87.2)	127	89	(70.1)	38	(29.9)	179	131	(73.6)	47	(26.4)
**CRC**	547	67	(12.2)	480	(87.8)	118	85	(72.0)	33	(28.0)	141	124	(87.9)	17	(12.1)
**Age**															
< 65	240	**28**	**(11.7)**	**212**	**(88.3)**^**0.736**^	44	**29**	**(66.9)**	**15**	**(34.1)**^**0.158**^	132	**96**	**(72.7)**	**36**	**(27.3)**^**0.656**^
> 65	285	**36**	**(12.6)**	**249**	**(87.4)**	60	**47**	**(73.1)**	**13**	**(26.9)**	46	**35**	**(76.1)**	**11**	**(23.9)**
**Sex**															
Male	312	**41**	**(13.1)**	**271**	**(86.9)**^**0.409**^	57	**37**	**(64.9)**	**20**	**(35.1)**^**0.084**^	120	**89**	**(74.2)**	**31**	**(25.8)**^**0.672**^
Female	214	**23**	**(10.7)**	**181**	**(89.3)**	54	**43**	**(79.6)**	**11**	**(20.4)**	59	**42**	**(71.2)**	**17**	**(28.8)**
**Tumor type**															
Adeno	491	**58**	**(11.8)**	**433**	**(88.2)**^**0.357**^	103	**76**	**(73.8)**	**27**	**(26.2)**^**0.266**^	90	**84**	**(93.3)**	**6**	**(6.7)**^**0.189**^
Others	56	**9**	**(16.1)**	**47**	**(83.9)**	15	**9**	**(60.0)**	**6**	**(40.0)**	19	**16**	**(84.2)**	**3**	**(15.8)**
**Tumor stage**															
0 and I	53	**11**	**(20.8)**	**42**	**(79.2)**^**0.043**^	9	**8**	**(77.8)**	**1**	**(22.2)**^**0.238**^	6	**6**	**(100.0)**	**0**	**(0.0)**^**0.450**^
II, III, and IV	449	**50**	**(11.1)**	**399**	**(88.9)**	98	**69**	**(70.4)**	**29**	**(29.6)**	103	**94**	**(91.3)**	**9**	**(8.7)**
**Tumor size**															
T0–T1	34	**4**	**(11.8)**	**30**	**(88.2)**^**0.938**^	5	**4**	**(80.0)**	**1**	**(20.0)**^**0.664**^	10	**10**	**(100.0)**	**0**	**(0.0)**^**0.320**^
T2–T4	483	**59**	**(12.2)**	**424**	**(87.8)**	100	**71**	**(71.0)**	**29**	**(29.0)**	99	**90**	**(90.9)**	**9**	**(9.1)**
**Regional lymph nodes metastasis**															
N = 0	269	**29**	**(10.8)**	**240**	**(89.2)**^**0.309**^	56	**40**	**(71.4)**	**16**	**(28.6)**^**0.897**^	63	**59**	**(93.7)**	**4**	**(6.3)**^**0.397**^
N > 1	248	**34**	**(13.7)**	**214**	**(86.3)**	51	**37**	**(72.5)**	**14**	**(27.5)**	46	**41**	**(89.1)**	**5**	**(10.9)**
**Distant metastasis**															
M = 0	396	**45**	**(11.4)**	**351**	**(88.6)**^**0.235**^	76	**55**	**(72.4)**	**21**	**(27.6)**^**0.788**^	92	**85**	**(92.4)**	**7**	**(7.6)**^**0.026**^
M > 1	102	**16**	**(15.7)**	**86**	**(84.3)**	28	**21**	**(75.0)**	**7**	**(25.0)**	49	**39**	**(79.6)**	**10**	**(20.4)**
**Differentiation grade**															
Well/moderate	479	**59**	**(21.4)**	**420**	**(87.7)**^**0.583**^	100	**73**	**(73.0)**	**27**	**(27.0)**^**0.577**^	91	**83**	**(91.2)**	**8**	**(8.8)**^**0.448**^
Poor	33	**3**	**(9.1)**	**30**	**(90.9)**	6	**5**	**(83.3)**	**1**	**(16.7)**	6	**6**	**(100.0)**	**0**	**(0.0)**
**Location**															
Cecum, appendix	47	**3**	**(6.4)**	**44**	**(93.6)**^**0.449**^	8	**6**	**(75.0)**	**2**	**(25.0)**^**0.891**^	5	**5**	**(100.0)**	**0**	**(0.0)**^**0.097**^
Ascending colon	88	**9**	**(10.2)**	**79**	**(89.8)**	10	**8**	**(80.0)**	**2**	**(20.0)**	21	**20**	**(95.2)**	**1**	**(4.8)**
Transverse colon	24	**2**	**(8.3)**	**22**	**(91.7)**	6	**4**	**(66.7)**	**2**	**(33.3)**	10	**7**	**(70.0)**	**3**	**(30.0)**
Descending colon	47	**9**	**(6.4)**	**38**	**(80.9)**	9	**5**	**(55.6)**	**4**	**(44.4)**	9	**8**	**(88.9)**	**1**	**(11.1)**
Sigmoid colon	161	**20**	**(12.4)**	**141**	**(87.6)**	27	**18**	**(66.7)**	**9**	**(33.3)**	31	**30**	**(96.8)**	**1**	**(3.2)**
Rectum	129	**13**	**(10.1)**	**116**	**(89.9)**	15	**11**	**(73.3)**	**4**	**(26.7)**	20	**19**	**(95.0)**	**1**	**(5.0)**
**Vascular invasion**															
No invasion	407	**49**	**(12.0)**	**358**	**(88.0)**^**0.458**^	59	**43**	**(72.9)**	**16**	**(27.1)**^**0.956**^					
Invasion	77	**7**	**(9.1)**	**70**	**(90.9)**	18	**13**	**(72.2)**	**5**	**(27.8)**					
**MSI**															
MSS	50	**3**	**(6.0)**	**47**	**(94.0)**^**0.419**^	37	**27**	**(73.0)**	**10**	**(27.0)**^**0.630**^					
MSI-L	6	**1**	**(16.7)**	**5**	**(83.3)**	5	**3**	**(60.0)**	**2**	**(40.0)**					
MSI-H	9	**0**	**(0.0)**	**9**	**(100.0)**	7	**4**	**(57.1)**	**3**	**(42.9)**					
**Tissue types**															
Malignant											109	**100**	**(91.7)**	**9**	**(8.3)**^**< 0.001**^
Metastasis											32	**24**	**(75.0)**	**8**	**(25.0)**
Benign tumor	9	**4**	**(44.4)**	**5**	**(55.6)**	9	**4**	**(44.4)**	**5**	**(55.6)**	10	**3**	**(30.0)**	**7**	**(70.0)**
Hyperplasia											8	**1**	**(12.5)**	**7**	**(87.5)**
Inflammation											10	**5**	**(50.0)**	**5**	**(50.0)**
Adjacent normal											11	**5**	**(45.5)**	**6**	**(54.5)**
Normal colon											8	**2**	**(25.0)**	**6**	**(75.0)**

These results were analyzed by the Pearson *X*^2^ test. Significant *p* values are indicated by superscripts

^a^For some categories, the number of samples (*n*) was lower than the overall number analyzed because clinical data were unavailable for those samples

^b^The *TMEM240* promoter methylation level in CRC tumors being 5-fold higher than in adjacent normal colorectal tissues was defined as hypermethylation

^c^The *TMEM240* expression level in CRC tumors being less than that of adjacent normal colorectal tissues was defined as low expression

### Low *TMEM240* mRNA expression in Taiwanese CRC patients and their associated poor prognoses

**Fig. 4:**
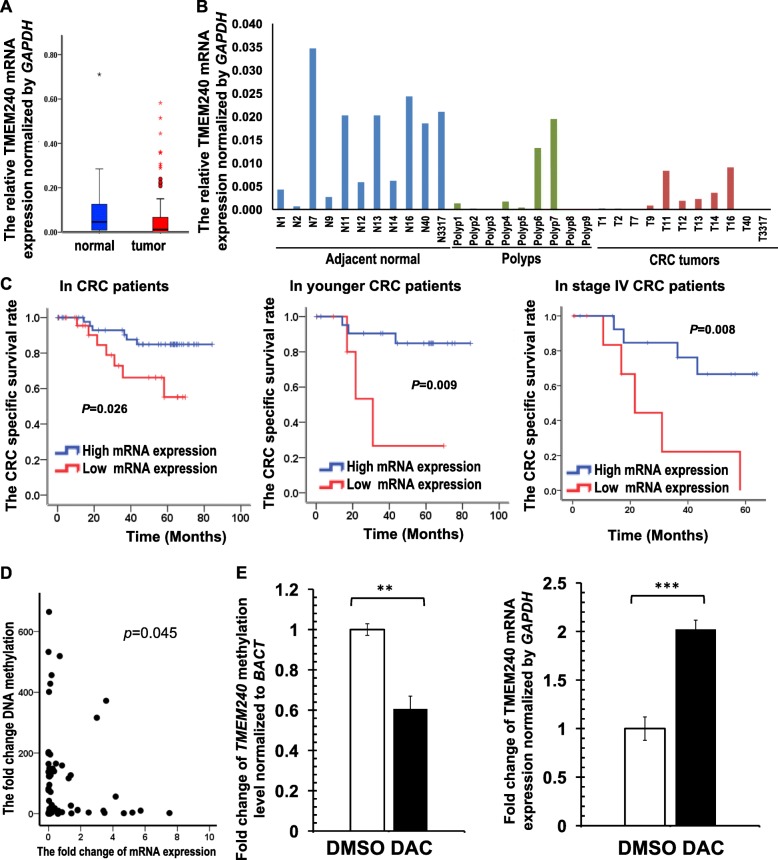
*TMEM240* mRNA expression levels are affected by DNA methylation and are associated with cancer-specific survival. **a** The box plot of *TMEM240* mRNA expression levels in tissues. **b** Representative figure of the *TMEM240* mRNA expression level determined by RT-qPCR in 10 adjacent normal colon tissues, nine polyps of tubular adenomas, and 10 colorectal carcinoma (CRC) tumors. **c** Kaplan-Meier survival curves were used to compare cancer-specific survival between CRC patients with low and those with high *TMEM240* mRNA expression. *TMEM240* was considered to have low expression when the expression level in CRC tumors was 5-fold lower than that in normal tissues. **d** Scatterplot of the fold change of *TMEM240* DNA methylation and mRNA expression levels in CRC tumors normalized to the adjacent normal tissues. **e** DNA methylation and mRNA expression were measured after treatment with decitabine (DAC). Fold changes in DNA methylation after treatment with DAC (left panel). Fold changes in *TMEM240* mRNA expression after treatment with DAC (right panel). Data are presented as the mean ± SD, **p ≤ 0.01, ***p ≤ 0.001. A *t* test was used to calculate group differences in all experiments. Experiments were performed with at least two biological duplicates and three technical replicates

We further analyzed *TMEM240* mRNA expression in 118 paired CRC tissues. The median *TMEM240* mRNA expression levels normalized by *GAPDH* were 0.0114 in CRC tumors and 0.0485 in adjacent normal tissues. (Fig. 4a, Figure S4, Table 1). In 72.0% (85/118) of tissue samples, *TMEM240* mRNA expression was lower in Taiwanese CRC tumor tissues than in normal colorectal tissues (Fig. 4a, Table 1). Loss of mRNA expression is especially higher in the cecum or appendix and ascending colon than in other locations (Table 1). Low *TMEM240* mRNA expression was found in four of the nine samples (44.4%) from patients with benign tubular adenoma (Fig. 4b, Table 1g). CRC patients with low mRNA expression of the *TMEM240* gene had relatively lower overall survival (OS) and cancer-specific survival (CSS) rates than CRC patients with high mRNA expression of *TMEM240* (*p* = 0.048 and 0.026, respectively) according to Kaplan-Meier survival curves and a log-rank test of 78 Taiwanese CRC patients, and this was especially true in female, stage IV and younger CRC patients (Fig. 4c, Figure S5, *p* = 0.021, 0.008, and 0.009, respectively). A Cox proportional hazards survival analysis further adjusted for sex, age, tumor stage, tumor location, and differentiation showed that *TMEM240* promoter hypermethylation and low mRNA expression were significantly and independently associated with OS, CSS, and recurrence-free survival (RFS) (Table 2, *p* = 0.028, 0.025, and 0.045, respectively).

**Table 2:** Cox proportional hazard model of clinical parameters and *TMEM240* mRNA expression level associated with Taiwan CRC patients

	Multivariate analysis			
Variable	HR	95% CI		*p* value
		Lower	Upper	
Overall survival				
Age	0.291	0.051	1.667	0.166
Sex	0.308	0.054	1.740	0.182
Stage	4.702	1.695	13.041	0.003
Location	2.970	0.985	8.951	0.053
Differentiation	0.599	0.030	11.897	0.737
DNA methylation	1.005	1.001	1.010	0.028
mRNA expression	0.196	0.055	0.704	0.012
Cancer specific survival				
Age	0.198	0.025	1.584	0.127
Sex	0.245	0.035	1.718	0.157
Stage	7.658	2.083	28.161	0.002
Location	4.055	1.257	13.083	0.019
Differentiation	0.463	0.017	12.307	0.646
DNA methylation	1.006	1.001	1.011	0.025
mRNA expression	0.142	0.035	0.587	0.007
Recurrence-free survival				
Age	0.360	0.088	1.473	0.155
Sex	0.358	0.095	1.345	0.128
Stage	9.385	3.249	27.112	< 0.000
Location	2.338	0.941	5.809	0.067
Differentiation	0.873	0.143	5.338	0.883
DNA methylation	1.004	1.000	1.008	0.045
mRNA expression	0.289	0.100	0.837	0.022

The DNA hypermethylation levels and mRNA expression levels of *TMEM240* were significantly negatively correlated according to a Spearman rank correlation analysis (*p* = 0.045, Fig. 4d). To determine whether hypermethylation of *TMEM240* was associated with mRNA expression, the suppression of *TMEM240* mRNA expression by hypermethylation was investigated via administration of the DNA demethylating drug decitabine (DAC) in the DLD-1 colon cancer cell line. Cells were treated with DMSO and DAC for 48 h. In the DAC group, the degree of methylation of *TMEM240* decreased to 60.57% of that in the DMSO group (*p* = 0.001, Fig. 4e), and *TMEM240* mRNA expression increased by 2.02-fold (*p* < 0.001, Fig. 4e), suggesting that hypermethylation of the *TMEM240* promoter is the main mechanism of *TMEM240* silencing.

### Low TMEM240 protein expression in south Korean and Chinese CRC patients

**Fig. 5:**
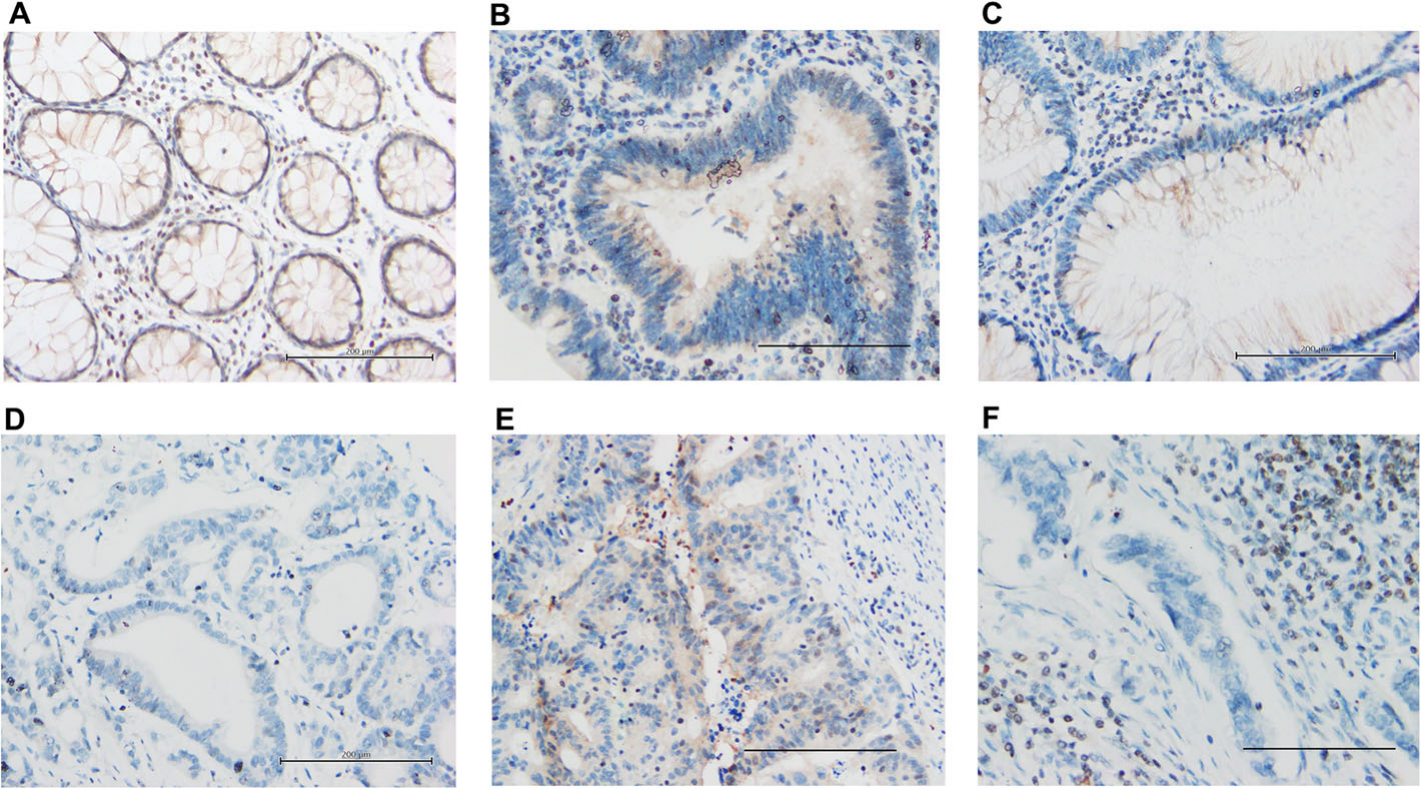
Representative figures for TMEM240 protein expression as analyzed by IHC. **a** Normal colon mucosa. **b** Hyperplastic polyp. **c** Benign colon tumor. **d** Colon cancer tissue with negative expression. **e** Colon cancer tissue with low expression. **f** Metastatic colon cancer tissue (original magnification, × 200). Scale bars indicate 200 μm

TMEM240 protein expression was analyzed in 109 tumors from CRC patients, 32 CRC metastatic tumors from patients, 10 benign tumors, eight hyperplastic tumors, 10 colon tissues with inflammation, 11 adjacent normal tissues, and eight normal colon tissues by IHC. The small, round, positive cells are lymphocytes, which serve as internal control cells (Fig. 5a). The TMEM240 protein was mainly expressed in the plasma membranes of the cells of the normal colonic mucosa (Fig. 5a). Note that positive TMEM240 protein staining was detected in 87.5% (7/8) and 70% (7/10) of the hyperplastic colon tumors and benign tumors, respectively (Fig. 5 b and c, Table 1). But it was deficiently or weakly expressed in the cytoplasm and nuclei of malignant CRC tumor cells (Fig. 5d–f). The TMEM240 protein level decreased in 91.7% (100/109) of tumors from CRC patients and in 75.0% (24/32) of the metastatic tumors from CRC patients but exhibited higher expression in 75.0% (6/8) of the normal colon tissues (Fig. 5, Table 1). The protein expression of TMEM240 was deficiently especially in the cecum or appendix, ascending, sigmoid colon, and rectum than in other locations (Table 1).

### *TMEM240* promoter hypermethylation and low mRNA expression in the CRC tissues from the TCGA dataset

**Fig. 6:**
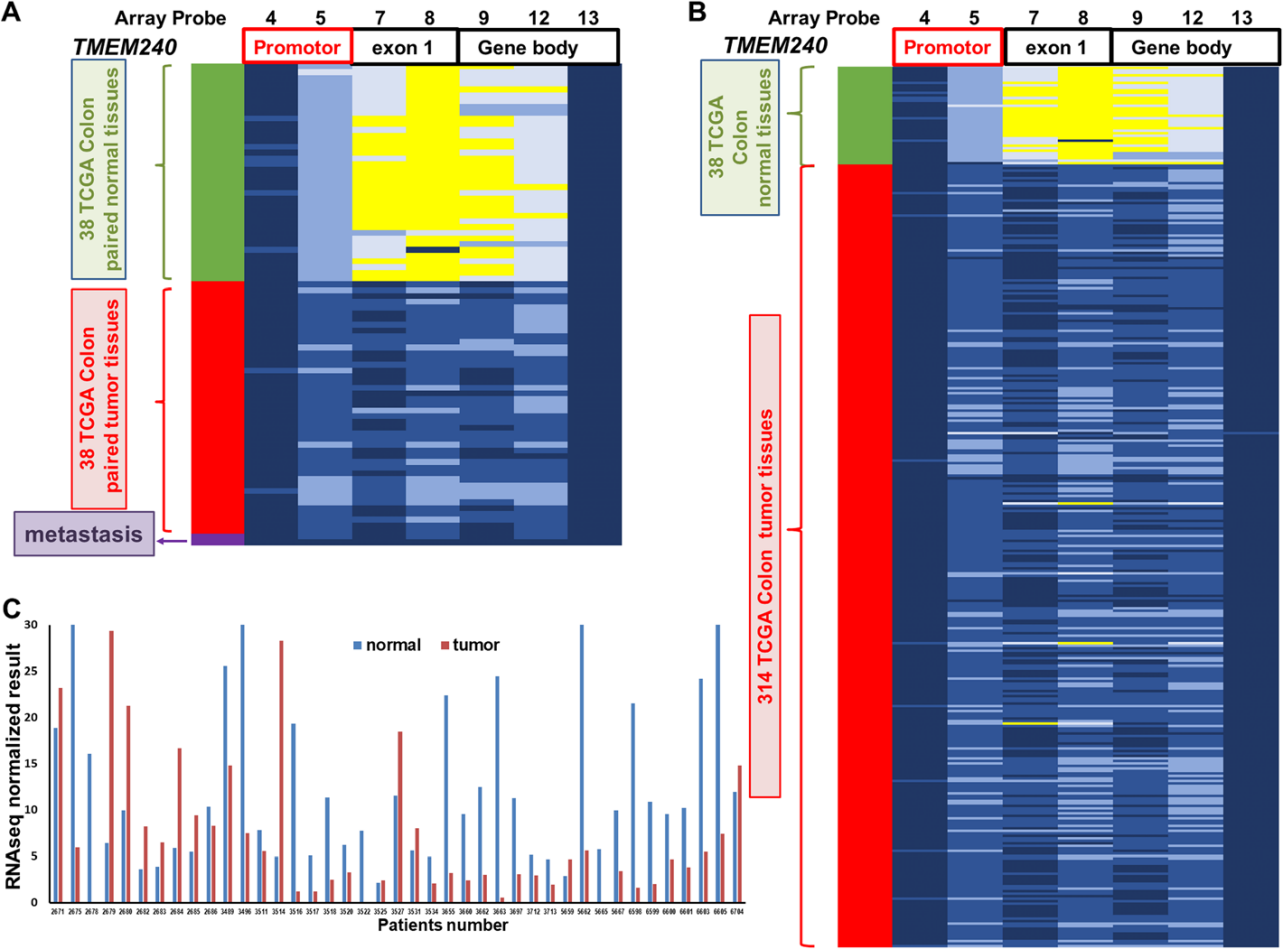
*TMEM240* DNA methylation and mRNA analysis from the TCGA dataset. Differentially methylated CpG sites in *TMEM240* were identified in **a** 38 adjacent normal colorectal tissues, 38 matched colorectal carcinoma (CRC) tumors, and **b** 314 CRC tumors by an Illumina Methylation 450K array-based assay. **c** RNA sequencing data for *TMEM240* in 41 adjacent normal colorectal tissues and 41 matched CRC tumors

To further determine alterations in *TMEM240* hypermethylation and mRNA expression in Western CRC patients, we first analyzed the TCGA data of 38 CRC tumors, 38 matched normal tissues, and 314 CRC tumor tissues that had been analyzed with an Illumina Infinium HumanMethylation450 BeadChip array and displayed the methylation levels with a heatmap. Again, the exon 1 region of *TMEM240* was hypermethylated in 314 colorectal tumor tissues (Fig. 6a, b). Analysis of RNA sequencing data from TCGA showed that *TMEM240* mRNA expression was markedly significantly reduced in 41 paired CRC tumor tissues compared to the matched normal colorectal tissues (*p* = 0.019, Fig. 6c). *TMEM240* promoter hypermethylation and low mRNA expression were found in 83.6% (245/293) and 85.3% (389/456) of CRC tumors, respectively (Table S2). The DNA hypermethylation levels and mRNA expression levels of *TMEM240* showed a significant negative correlation by Spearman rank correlation analysis (*p* = 0.045). Hypermethylation of *TMEM240* was associated with older CRC patients and patients without the kras mutation (*p* = 0.008 and 0.038, respectively, Table S2). Low expression of *TMEM240* mRNA was associated with CRC patients without regional lymph node metastasis (*p* = 0.028, Table S2). However, using Kaplan-Meier survival curves and a log-rank test to analyze whether the low expression of *TMEM240* is associated with poor survival in the TCGA data, the result shows no significant correlation between low expression of *TMEM240* and survival (*p* = 0.163).

## Discussion

Aberrant promoter hypermethylation of CpG islands associated with TSGs can cause transcriptional silencing and contribute to tumorigenesis. Advancements in detection technology have reduced CRC death rates in several Western countries [28]. Therefore, developing biomarkers for early detection and intervention can improve patient outcomes. Recent studies reported that several TSGs are often methylated in the tumorigenesis that involves the transformation of normal colonic epithelium to adenocarcinoma [29]. In the present investigation, highly methylated CpG sites in the promoter and exon 1 regions of *TMEM240* were identified using genome-wide methylation arrays. A QMSP confirmed *TMEM240* hypermethylation in CRC tumor tissues compared to normal tissues. In the Asian and TCGA cohorts, hypermethylation of the promoter region of *TMEM240* in more than 80% of tumors was consistently found regardless of age, sex, tumor type, stage, tumor size, regional lymph node metastasis, or distant metastasis. Low expression of the *TMEM240* protein was found in most Korean and Chinese CRC patients. Moreover, patients with low *TMEM240* mRNA expression had poor OS, CSS, and RFS, especially in young and stage IV CRC patients. We speculate that DNA hypermethylation induces lower expression of TMEM240 and then subsequently induces poor survival. However, the *TMEM240* expression was not associated with survival in TCGA data set, although *TMEM240* DNA hypermethylation and low mRNA expression were commonly observed in TCGA. Whether low expression of *TMEM240* was correlated with survival, only in Asian CRC patients need to be further studied in more specimens from Asian and Western countries using the same experimental technique.

Low expression of the TMEM240 protein was also observed in 50.0% of patients with inflammatory bowel diseases (IBDs) (Table 1). Individuals with IBDs have a 3~5-fold increased risk of CRC, as reported in a previous study [24]. Whether TMEM240 also plays an important role in IBDs during CRC tumorigenesis is worthy of further investigation. Although the anti-TMEM240 antibody used on the Human Protein Atlas website (https://www.proteinatlas.org/ENSG00000205090-TMEM240) is the same as the one we used (Sigma-Aldrich, HPA066721), it does not detect TMEM240 in normal colon tissues but does detect it in Purkinje cells in the human cerebellum. We used normal cerebellum Purkinje cells confirm the positive signal. Because *TMEM240* mRNA expression was successfully stably detected at a low level in almost all normal colorectal tissues, we tried to find better antibody conditions for protein detection. Therefore, we also tested the antibody at different dilutions. We found that the staining was obvious in the membrane compartment of normal colorectal tissues when the antibody was diluted at a 1:35 ratio but still exhibited no staining in colorectal tumor parts. The immunoexpression in CRC tissues was also consistent with our mRNA expression results. Therefore, we suggested that a lower dilution could be better for detecting lower levels of TMEM240 protein expression.

The *TMEM240* gene encodes a transmembrane domain-containing protein found in the brain and cerebellum. The protein structure in UniProtKB/Swiss-Prot showed that the protein has two transmembrane protein regions located at amino acids 5~25 and 90~110. We observed that the TMEM240 protein was mainly expressed in the plasma membranes of the cells of the normal colon mucosa but was deficiently or weakly expressed in the cytoplasm and nuclei of malignant CRC tumor cells (Fig. 5). The data is similar to the result of mRNA expression level. Whether the distribution of TMEM240 protein across cell compartments, ranging from plasma membranes to the cytoplasm and nuclei, and the accompanying biological functional loss are involved in tumorigenesis needs to be further investigated. The precise protein location will be worth of further investigation. The single-cell RNA-sequencing or single-cell proteomic mass spectrometry approaches would be worth to explore specific colon cellular gene and protein expression and localization [30, 31].

The biological role and function of the TMEM240 protein are unclear. In vitro cell model studies have suggested that both the knockdown and overexpression of TMEM240 can repress cell proliferation. Flow cytometry revealed that *TMEM240* knockdown induced a decrease in the proportion of cells in the G_1_ phase and an increase in cells in the G_2_/M phase. However, an opposite trend was observed when TMEM240 was overexpressed (Fig. 2). These data suggest that TMEM240 reduces 4.28%~6.33% of the cell proportion through cell cycle arrest and probably participates in other mechanisms, such as apoptosis, autophagy, and cellular differentiation. Whether TMEM240 protein is a potential anticancer target is worthy of investigation. DAC treatment increased *TMEM240* mRNA expression through *TMEM240* promoter demethylation, suggesting that hypermethylation of the *TMEM240* promoter is the principle mechanism behind *TMEM240* silencing. In addition, DAC sensitizes CRC cells to topoisomerase inhibitors (irinotecan, etoposide, doxorubicin, and mitoxantrone) [32]. Whether DAC, DNA methyltransferase inhibitors, or other DNA demethylating agents exert relevant anticancer effects through inducing TMEM240 expression is worthy of further investigation [33].

*TMEM240* DNA methylation and mRNA expression were analyzed in 556 Taiwanese patients. TMEM240 protein expression was analyzed in 108 South Korean patients and 83 Chinese patients. Our study also analyzed the DNA methylation and mRNA expression of *TMEM240* in 456 Western TCGA patients. The results support the idea that hypermethylation and decreased expression of *TMEM240* are common characteristics in patients from both Asian and Western countries. Note that hypermethylation of *TMEM240* was found in 87.2% of Taiwanese CRC tissues, which is much higher than that found for hypermethylation of SEPT9 (60.92%) in Taiwanese CRC patients. The data suggest that hypermethylation of *TMEM240* could be a superior early predictive biomarker in Asian CRC patients. In addition, hypermethylation of *TMEM240* was found in 55.6% of patients with polyp tubular adenomas, supporting hypermethylation of *TMEM240* as an early-onset indicator of CRC. Although the sensitivity of *TMEM240* (52.0%) for stages I~III CRC was less than that of *SEPT9* (68%, stages I~III), the sensitivity was analyzed with 3.5 mL of plasma. However, our study was limited by the fact that the available volume of plasma for the free methylated *TMEM240* analysis was 200 μL, which is much less than the 3.5 mL of plasma used for the analysis of free methylated *SEPT9*. Approximately 90% of CRC patients exhibit *TMEM240* hypermethylation, and the detection of circulating methylated *TMEM240* will still improve early diagnosis and provide a noninvasive method to monitor the residual tumor burden after treatment. Therefore, free methylated *TMEM240* is a potential biomarker for the early prediction of CRC, encouraging us to further enroll additional patients and perform a study with more plasma samples. However, circulating methylated *TMEM240* showed a 28.6% false-positive rate for early prediction. Improving the specificity of circulating cell-free DNA extraction to decrease residual genomic DNA during extraction may decrease the false-positive rate. The addition of other genes with altered methylation levels, such as *ZNF625*, *TMEM240*, *SLC6A15*, and *MPPED2*. The *ZNF625*, *SLC6A15*, and *MPPED2* had been also found hypermethylation in tumors of CRC patients in previous studies [23–25]. However, whether the hypermethylation of those genes can be detected in ccfDNA is worth further investigation. In addition, the hypermethylation of the *ZNF625*, *TMEM240*, *SLC6A15*, and *MPPED2* genes used in the predictive model may largely increase the sensitivity, improve the accuracy, and decrease the false-positive rate.

Interestingly, *TMEM240* hypermethylation was also found in other cancers of the digestive system, such as esophageal and liver cancers. The identification of *TMEM240* hypermethylation using Infinium HumanMethylation450 BeadChips was also reported in the US hepatocellular carcinoma patients [34]. We also analyzed other cancers, such as lung cancer, and a lower aberrant frequency was found. These data imply that *TMEM240* hypermethylation mainly occurs in the gastrointestinal tract, especially in the regions involved in food transport, including the esophagus, stomach, colon, and rectum. Whether aberrant hypermethylation of *TMEM240* is associated with eating habits is also worthy of further study. In addition to its use in CRC, *TMEM240* hypermethylation may also be an early-onset indicator in gastrointestinal tract cancer.

## Conclusions

The current study revealed that *TMEM240* hypermethylation was an early-onset CRC indicator. Thus, circulating free methylated *TMEM240* is associated with poor prognosis and can be applied as a noninvasive biomarker for early detection and early recurrence prediction.

## Methods

### Patients and tissue, plasma collection

**Fig. 7:**
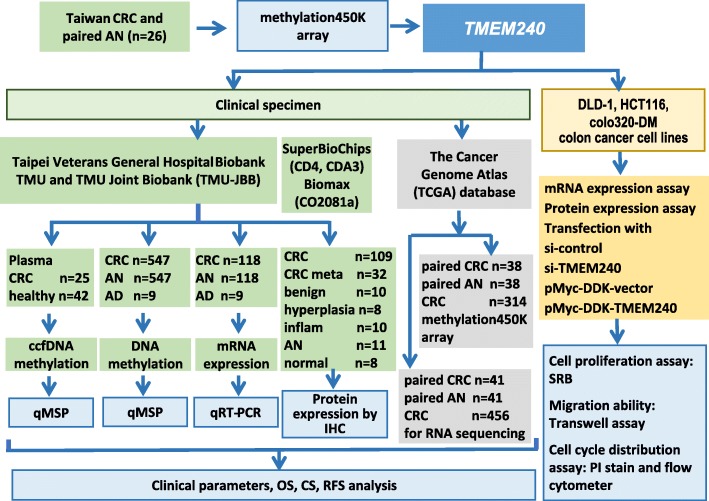
Flowchart of the study design, datasets, and specimens used. For each step, the sample types and number of samples used for the analyses are indicated. CRC, colorectal cancer; AN, adjacent normal; AD, benign adenoma; inflam, inflammation; normal, normal tissues; ccfDNA, circulating cell-free DNA; QMSP, quantitative methylation-specific PCR; qRT-PCR, quantitative reverse-transcription PCR; IHC, immunohistochemistry; methylation450K array, Illumina Infinium HumanMethylation450 BeadChip array; OS, overall survival; CS, cancer-specific survival; RFS, recurrence-free survival; SRB, sulforhodamine B assay; PI, propidium iodide staining

In total, 547 human CRC tissues, 547 paired adjacent normal colorectal tissues, nine polyp (tubular adenoma) tissues, and 67 plasma samples were obtained from the Taipei Medical University (TMU) Joint Biobank and Taipei Veterans General Hospital Biobank (Fig. 7). Before clinical data and sample collection, written informed consent was obtained from all patients. Patients undergoing preoperative chemoradiotherapy or an emergent operative procedure, those who died within 30 postoperative days, or those with evidence of familial adenomatous polyposis or Lynch syndrome were excluded from this study. Sections of cancerous tissues and corresponding noncancerous tissues were reviewed by a senior gastrointestinal pathologist. The adjacent nontumor part was collected from the colonic mucosa 10 cm proximal from the main tumor. Clinical data on sex, personal and family medical histories, tumor location, TNM tumor stage, tumor differentiation, microsatellite instability (MSI), pathological features, and follow-up conditions, which were prospectively collected, were obtained from the TMU Joint Biobank and Taipei Veterans General Hospital Biobank. Following surgery, patients were monitored every 3 months for the first 2 years and semiannually thereafter.

### Genomic DNA, circulating cell-free (cf) DNA, and RNA extraction

Genomic DNA from matched pairs of primary tumor tissues and adjacent colorectal tissues from the same patient was extracted using the QIAamp DNA Mini Kit (Qiagen, Bonn, Germany, cat. no. 51306) according to the manufacturer’s instructions. For RNA extraction, tumor and normal specimens were immediately frozen after surgical resection and stored at − 80 °C. Total mRNA was extracted using the RNeasy Plus Mini Kit (Qiagen, cat. no. 74134) according to the manufacturer’s instructions. ccfDNA was extracted from plasma. The ccfDNA of 26 plasma samples was extracted using a MagMAX Cell-Free DNA Isolation Kit according to the manufacturer’s recommended protocol (Thermo Fisher Scientific, Austin, TX, USA) [35, 36]. The ccfDNA in 200 μL of plasma for each of the 41 patients was extracted using a QIAamp MinElute Virus Spin Kit according to the manufacturer’s recommended protocol [37].

### Quantitative reverse-transcription polymerase chain reaction (qRT-PCR)

To measure *TMEM240* mRNA expression, real-time RT-PCR was performed with a LightCycler 480 (Roche Applied Science, Mannheim, Germany). Real-time PCR was performed using the LightCycler 480 Probe Master Kit (Roche Applied Science, Indianapolis, IN, USA, cat. no. 04707494001) with specific primers and a corresponding universal library probe (Roche Applied Science) according to the manufacturer’s instructions. The glyceraldehyde 3-phosphate dehydrogenase (GAPDH) gene was used as a reference gene. PCR conditions were as follows: preincubation, 95 °C for 10 min; and amplification, 95 °C for 10 min and 60 °C for 10 min, for a total of 45 cycles. Normalized gene expression values obtained using the LightCycler Relative Quantification software (vers. 1.5, Roche Applied Science) were then compared to those of the control group. *TMEM240* mRNA expression was considered low if the mRNA expression level of *TMEM240* relative to GAPDH was 0.5-fold lower in colorectal tumor tissues than in paired normal colorectal tissues. The primers and probes used in the RT-PCR are listed in Supplementary Table S1.

### TaqMan quantitative methylation-specific PCR (QMSP)

After bisulfite conversion of genomic DNA using the EpiTect Fast DNA Bisulfite Kit (Qiagen, Bonn, Germany, cat. no. 59826) according to the manufacturer’s recommended protocol, the DNA methylation level of *TMEM240* was measured using TaqMan QMSP with a LightCycler 480 (Roche Applied Science). The QMSP was performed with the SensiFAST™ Probe No-ROX Kit (Bioline, London, UK, cat. no. BIO-86020) with specific primers and a methyl-TaqMan probe for *TMEM240*. Normalized DNA methylation values, which were calibrated to the control group, were obtained using the LightCycler Relative Quantification software (vers. 1.5, Roche Applied Science). The *beta-actin* (*ACTB*) gene was used as a reference gene. Primers and probes for *TMEM240* methylation detection were designed for the junction between the promoter and exon 1 regions. The relative *TMEM240* DNA methylation level was determined with a methylated specific *TMEM240* probe and primer (with 7 CpG sites) and normalized to ACTB (without a CpG site). ACTB can work as total genomic DNA content control. Only when all 7 CpGs of *TMEM240* genes show methylation at the same time can the PCR be successfully amplified and detected. In 7 total CpG sites, 3 CpGs were detected by forward primer, one CpG was detected by probe, and 3 CpGs were detected by reverse primer. *TMEM240* was considered hypermethylated when the methylation level of *TMEM240* relative to that of the *ACTB* gene was at least 2-fold higher in the colorectal tumor compared to the paired normal colorectal tissue sample. The specificity of *TMEM240* methylation end products was confirmed by bisulfite sequencing. The primers and probes used in the QMSP are listed in Supplementary Table S1.

### Genome-wide methylation analysis

The genome-wide methylation analysis of 26 paired CRC tissues and corresponding noncancerous colon tissues was performed using the Illumina Infinium HumanMethylation450 BeadChip array (Illumina, San Diego, CA, USA), as previously described [15, 17]. The array contains more than 450,000 methylation sites and provides genome-wide coverage of gene regions and CpG island coverage, including 99% of RefSeq genes. Bisulfite conversion was performed for 500 ng of genomic DNA using the EpiTect Fast DNA Bisulfite Kit (Qiagen, cat. no. 59826), according to the manufacturer’s instructions. Methylation scores for each CpG site are represented as “beta” values ranging from 0 (unmethylated) to 1 (fully methylated), which were calculated by determining the ratios of methylated signal intensities to the sums of the methylated and unmethylated signal outputs.

### Cell lines, cell culture, and drug treatment

The DLD-1, HCT116, and colo320-DM cell lines used in this study are CRC cells obtained from the Bioresource Collection and Research Center (http://www.bcrc.firdi.org.tw/). The DLD-1 and colo320-DM were cultured in UltraCulture serum-free medium (Lonza, Walkersville, MD, USA, cat. no. 12–725F). HCT116 cells were cultured with 2.5% human platelet lysate (hPL, Compass Biomedical, MA, USA) and 1% penicillin/streptomycin (Thermo Fisher Scientific). To demethylate *TMEM240*, DLD-1 cells were treated with dimethyl sulfoxide (DMSO) or the demethylation agent decitabine (DAC, Sigma-Aldrich, St. Louis, MO, USA). After treatment, DNA and RNA were extracted, and methylation and expression levels were analyzed. DAC was dissolved in DMSO.

### Immunohistochemistry (IHC) assay

Three sets of tissue microarrays of colon tissues and CRC tissues were created to analyze TMEM240 protein expression. Two sets of tissue microarrays of CRC were purchased from SuperBioChips Laboratories (cat. nos. CD4 and CDA3; Seoul, South Korea). A third tissue microarray was purchased from Biomax (cat. no. CO2081a; US Biomax, Rockville, MD, USA). Tissue microarrays were composed of colorectal tumor tissues obtained from 105 South Korean samples of CRC tumors and 104 Chinese samples, including 45 CRC tumors, 32 CRC metastatic tumors, 10 benign tumors, 8 hyperplastic tumors, 10 colon tissues with inflammation, 11 adjacent normal tissues, and 8 normal colon tissues (Fig. 7). The pathologic diagnoses of these cases were microscopically confirmed by two researchers. IHC staining with an anti-TMEM240 antibody (1:35, Sigma-Aldrich, HPA066721) was performed using an iView DAB detection kit (Ventana, Tucson, AR, USA) on a BenchMark XT autostainer. This assay included both positive and negative controls. The researchers who evaluated the IHC staining results were blinded to the clinical follow-up data. The intensity of TMEM240 expression was identified semiquantitatively as no expression, low expression (weaker than or equal to the expression intensity of normal colon epithelium), or high expression (stronger than the expression intensity of normal colon epithelium).

### Immunoblotting analyses

For Western blotting assays, the cells were lysed on ice in radioimmunoprecipitation buffer (0.05 M Tris-HCl [pH 7.4], 0.15 M NaCl, 0.25% deoxycholic acid, 1% IGEPAL CA-630, and 1 mM ethylenediaminetetraacetic acid). The lysates were centrifuged at 13,000 rpm at 4 °C for 10 min. The protein extracts were solubilized in sodium dodecyl sulfate (SDS) gel loading buffer (60 mmol/L Tris base, 2% SDS, 10% glycerol, and 5% β-mercaptoethanol). Samples containing equal amounts of protein (40 μg) were separated on an 8% SDS-polyacrylamide gel by using electrophoresis and electroblotted onto Immobilon-P membranes (Millipore, Bedford, MA, USA) in transfer buffer. Immunoblotting was performed using an anti-DDK monoclonal antibody (1:3,000, OriGene, Rockville, MD, US) that was raised against the Myc-DDK-hTMEM240 protein. β-actin (1:5,000, GeneTex, Texas, USA) was used as an internal control.

### Plasmid extraction, confirmation, and purification

Plasmid DNA was extracted using a Geneaid™ Midi Plasmid Kit (Geneaid Biotech, cat. no. PI025) according to the manufacturer’s instructions. Plasmid DNA was then subjected to a preliminary length analysis by restriction enzyme digestion and sequenced to confirm accurate production. The plasmid concentration was measured with a NanoDrop 2000C ultra-microwave-length spectrometer (Thermo Fisher Scientific, Wilmington, DE, USA) and stored at − 20 °C until further usage.

### cDNA expression construct, RNAi, and transfection

The pMyc-DDK-hTMEM240 and pMyc-DDK vector control were used to transfect DLD-1 cells by the Neon® electrotransfection systems and reagent sets (Thermo Fisher Scientific, Cat. No. MPK1025) according to the manufacturer’s instructions. Briefly, 5.6 × 10^6^ cells were seeded and transfected with 1 μg of plasmid at 1100 V for 20 ms. Immediately, cells were transferred to culture medium and incubated. The two si-TMEM240 RNAi molecules that target exon 4 (s50534) and exon 3 (s50536) were obtained from Thermo Fisher Scientific (Cat. No. 4392420).

### Cell cycle distribution assay

The cell cycle distribution was studied by flow cytometry. DLD-1 cells (10^6^) were trypsinized and fixed overnight with 80% ethanol at − 20 °C. Fixed cells were stained with a solution containing 20 μg/mL of propidium iodide, 200 μg/mL of RNase A, and 0.1% Triton X-100 for 30 min in the dark. Cell cycle distributions were studied with a FACSCanto II flow cytometer (BD Biosciences, San Jose, CA, USA), and calculations were performed using the ModFIT LT vers. 2.0 software (Verity Software House, USA).

### Transwell assay

A transwell assay was used to study cell migration. The transwell separates the upper and lower wells with a semipermeable membrane (Falcon) with a pore size of 8 μm. Approximately, 2 × 10^4^ treated and untreated DLD-1 cells were seeded into the upper layer; 300 μL of serum-free Dulbecco’s Modified Eagle Medium (DMEM)/F12 was added as culture medium, and 800 μL of the serum-containing culture medium was added as a chemical attractant to the lower layer. After 16 h of incubation, cells that had passed through the membrane were washed twice with phosphate-buffered saline (PBS), fixed, and stained with 1% crystal violet/double-distilled (dd) H_2_O for 60 min at room temperature. Five random areas were photographed with a microscope (Nikon, TokyoJapan), followed by analysis with the ImageJ software to quantify the number of cells.

### Sulforhodamine B (SRB) assay

An SRB assay was used to determine the cell growth rate. DLD-1 cells were seeded in 96-well plates at a density of 8000 cells/well and incubated for 24 h. Cells were fixed with 10% trichloroacetic acid for 10 min. After staining with SRB for 30 min, excess dye was removed by washing the cells five times with 1% acetic acid. Cell growth was assessed using a microplate reader to determine the absorbance of the SRB solution at 515 nm. Growth inhibition rates were calculated as follows:

Cell growth inhibition rate (%) = 100 − [(Ti − Tz)/(C − Tz)] × 100 (Ti ≥ Tz);

where Ti = OD of the inhibitor sample, Tz = OD of basal cells, and C = OD of the control. Images were acquired using an inverted microscope (EVOS, AMG, USA) at the indicated time points. Cells were counted using the ImageJ software.

### Statistical analysis

Pearson’s *X*^2^ test was used to analyze *TMEM240* hypermethylation, mRNA, and protein expression in patients with colorectal cancer, and correlations with various clinical parameters, including age, sex, cancer type, stage, degree of differentiation, and degree of infringement, were assessed. All statistical analyses were performed using the SPSS software (SPSS, Chicago, IL, USA). Overall survival, cancer-specific survival, and recurrence-free survival curves were calculated and analyzed using the Kaplan-Meier method and multivariate Cox proportional hazards regression analysis. Comparisons of the survival curves with a log-rank test that resulted in a *p* of < 0.05 were considered statistically significant.

## Supplementary information

**Additional file 1: Figure S1.** Differentially methylated CpG heatmap of *TMEM240* in paired colon cancer, esophageal cancer, gastric cancer, pancreatic cancer, rectal cancer, and liver cancer tissues. Methylation levels (average β values) at differentially methylated loci were identified using an Illumina Methylation 450K array-based assay. **Figure S2**. *TMEM240* mRNA expression was analyzed in two colon cancer cell lines and two human normal colon tissues. **Figure S3**. Representative figures of *TMEM240* methylation levels determined by QMSP in 78 adjacent normal colon tissues and 78 CRC tumors. Experiments were performed with three technical replicates. **Figure S4**. Representative figures of *TMEM240* mRNA expression determined by gene specific probe based real-time RT-PCR in 21 adjacent normal colon tissues and 21 CRC tumors. Experiments were performed with three technical replicates. Data are presented as the mean ± SD, * *p* ≤ 0.05, ** *p* ≤ 0.01, *** *p* ≤ 0.001. A *t*-test was used to calculate group differences in all experiments. Experiments were performed with at least two biological duplicates and three technical replicates. **Figure S5**. The Kaplan-Meier survival curves for (A) overall survival in Taiwanese colorectal carcinoma (CRC) patients and (B) cancer-specific survival in Taiwanese females are shown. TMEM240 was considered to have low expression when the expression level in CRC tumors was 5-fold lower than that in normal tissues. **Table S1**. *TMEM240* mRNA expression and promoter hypermethylation in relation to the clinical parameters of the TCGA CRC dataset. **Table S2**: List of primers sequences and their reaction conditions used in the present study.

## Data Availability

The data generated in this study are available from the corresponding author upon reasonable request.

## Abbreviations

ACTB: Beta-actin
ccfDNA: Circulating cell-free DNA
cmDNA: Circulating methylated DNA
CRC: Colorectal cancer
GAPDH: Glyceraldehyde 3-phosphate dehydrogenase
OS: Overall survival
QMSP: Quantitative methylation-specific polymerase chain reaction
RT-qPCR: Reverse-transcription quantitative polymerase chain reaction
SCA21: Spinocerebellar ataxia 21
SEPT9: Septin 9
SRB: Sulforhodamine B
TCGA: The Cancer Genome Atlas
TMEM240: Transmembrane protein 240
TSG: Tumor suppressor gene
TMU: Taipei Medical University
