# Supplementary material for: Hypermethylation and decreased expression of TMEM240 are potential early-onset biomarkers for colorectal cancer detection, poor prognosis, and early recurrence prediction
Source: Clin Epigenetics. 2020 May 12;12:67. doi: 10.1186/s13148-020-00855-z (PMC7218647; doi:10.1186/s13148-020-00855-z)
Supplement: Supplementary file 1 — Additional file 1: Figure S1. Differentially methylated CpG heatmap of TMEM240 in paired colon cancer, esophageal cancer, gastric cancer, pancreatic cancer, rectal cancer, and liver cancer tissues. Methylation levels (average β values) at differentially methylated loci were identified using an Illumina Methylation 450K array-based assay. Figure S2. TMEM240 mRNA expression was analyzed in two colon cancer cell lines and two human normal colon tissues. Figure S3. Representative figures of TMEM240 methylation levels determined by QMSP in 78 adjacent normal colon tissues and 78 CRC tumors. Experiments were performed with three technical replicates. Figure S4. Representative figures of TMEM240 mRNA expression determined by gene specific probe based real-time RT-PCR in 21 adjacent normal colon tissues and 21 CRC tumors. Experiments were performed with three technical replicates. Data are presented as the mean ± SD, * p ≤ 0.05, ** p ≤ 0.01, *** p ≤ 0.001. A t-test was used to calculate group differences in all experiments. Experiments were performed with at least two biological duplicates and three technical replicates. Figure S5. The Kaplan-Meier survival curves for (A) overall survival in Taiwanese colorectal carcinoma (CRC) patients and (B) cancer-specific survival in Taiwanese females are shown. TMEM240 was considered to have low expression when the expression level in CRC tumors was 5-fold lower than that in normal tissues. Table S1. TMEM240 mRNA expression and promoter hypermethylation in relation to the clinical parameters of the TCGA CRC dataset. Table S2: List of primers sequences and their reaction conditions used in the present study. [file 13148_2020_855_MOESM1_ESM.docx]

**Hypermethylation and Decreased Expression of *TMEM240* are Potential Early-Onset Biomarkers for Colorectal Cancer Detection, Poor Prognosis and Early Recurrence Prediction**

**Supplementary figures**

**
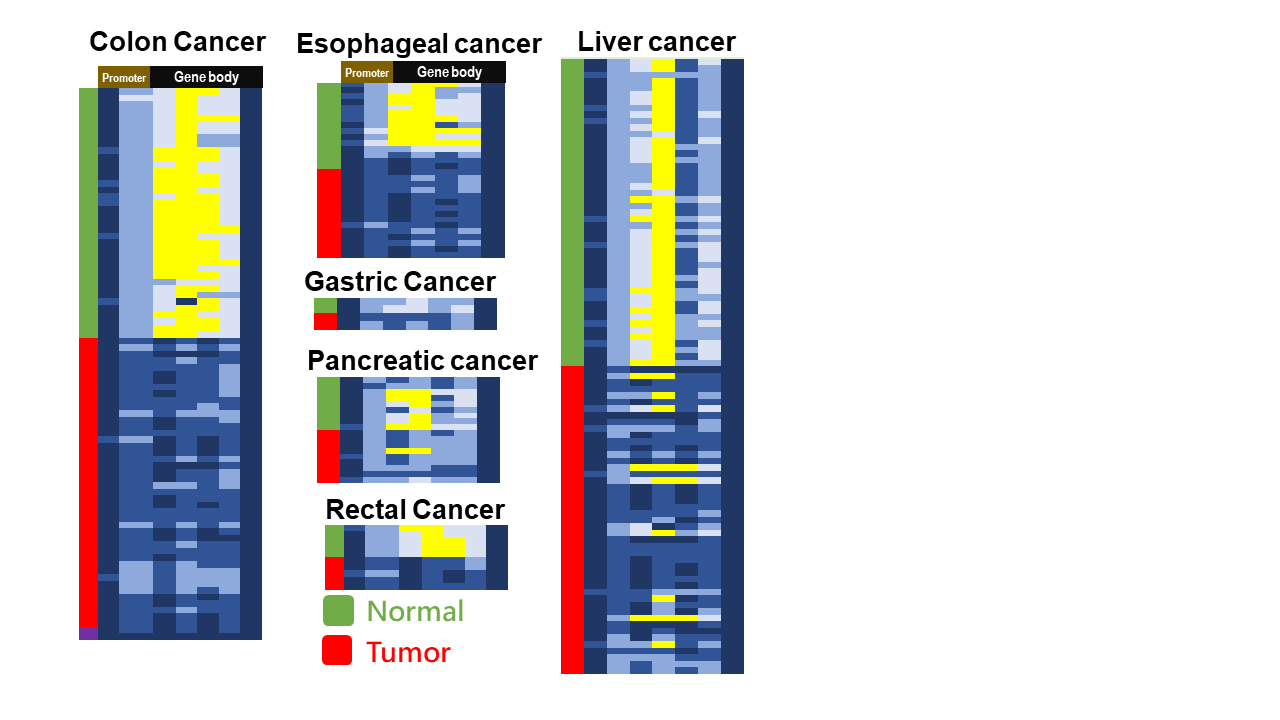
**

**Figure S1.** Differentially methylated CpG heatmap of *TMEM240* in paired colon cancer, esophageal cancer, gastric cancer, pancreatic cancer, rectal cancer, and liver cancer tissues. Methylation levels (average β values) at differentially methylated loci were identified using an Illumina Methylation 450K array-based assay.

**
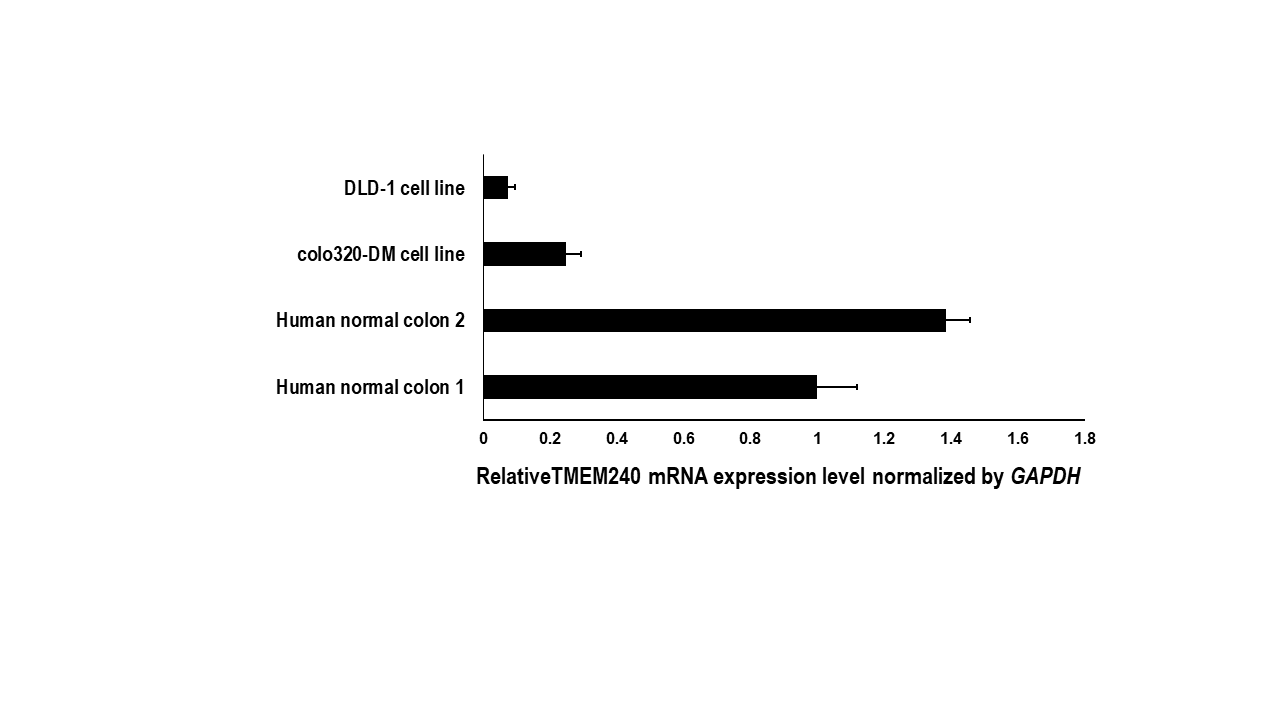
**

**Figure S2.** *TMEM240* mRNA expression was analyzed in two colon cancer cell lines and two human normal colon tissues.


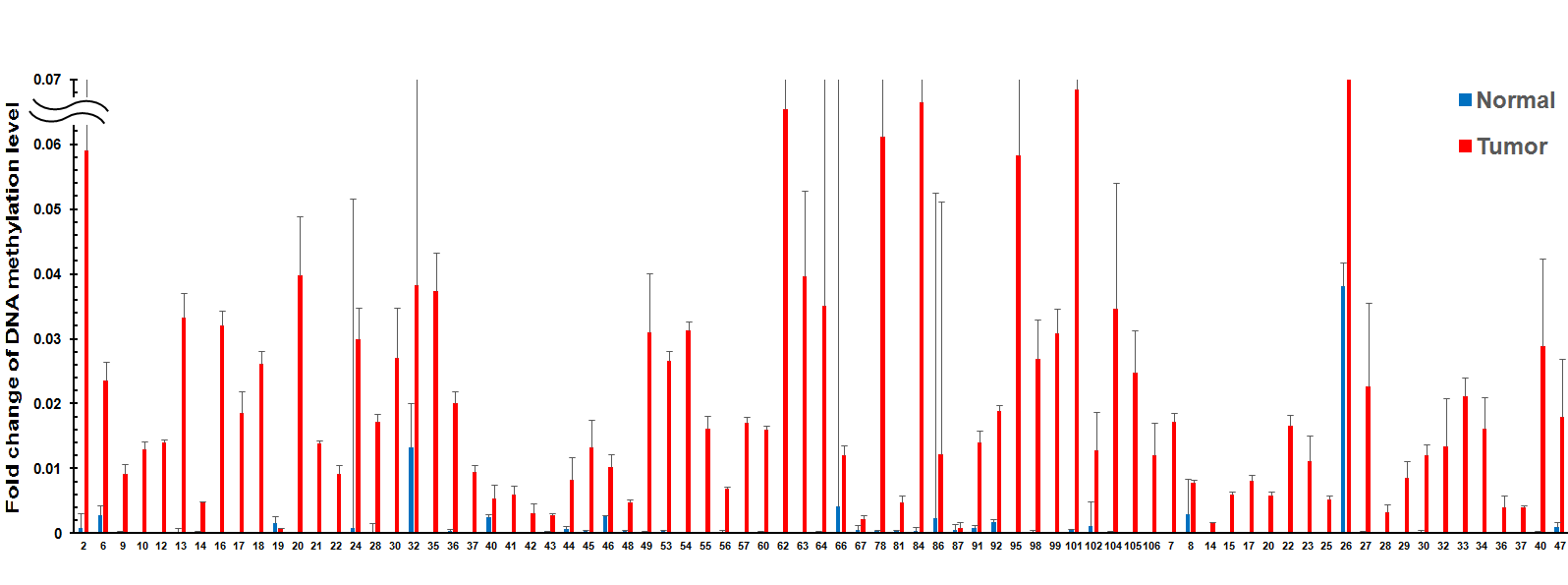


**Figure S3.** Representative figures of *TMEM240* methylation levels determined by QMSP in 78 adjacent normal colon tissues and 78 CRC tumors. Experiments were performed with three technical replicates.


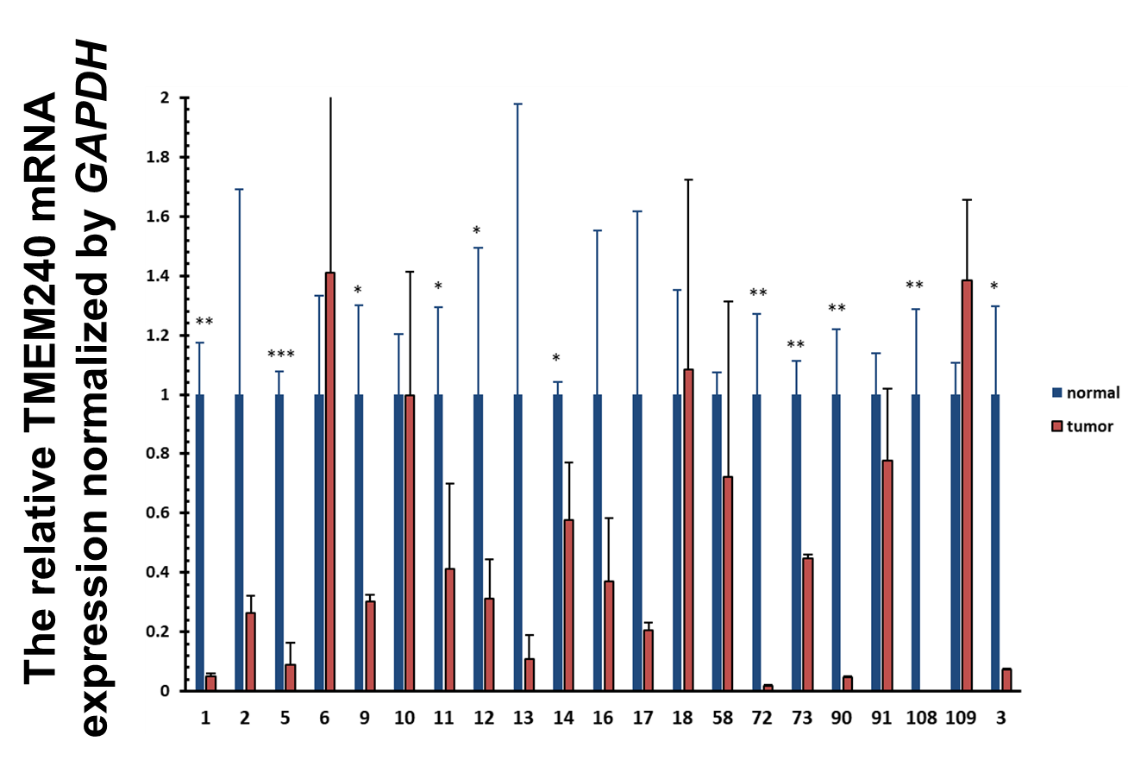


**Figure S4.** Representative figures of *TMEM240* mRNA expression determined by gene specific probe based real-time RT-PCR in 21 adjacent normal colon tissues and 21 CRC tumors. Experiments were performed with three technical replicates. Data are presented as the mean ± SD, * *p* ≤ 0.05, ** *p* ≤ 0.01, *** *p* ≤ 0.001. A *t*-test was used to calculate group differences in all experiments. Experiments were performed with at least two biological duplicates and three technical replicates.

**
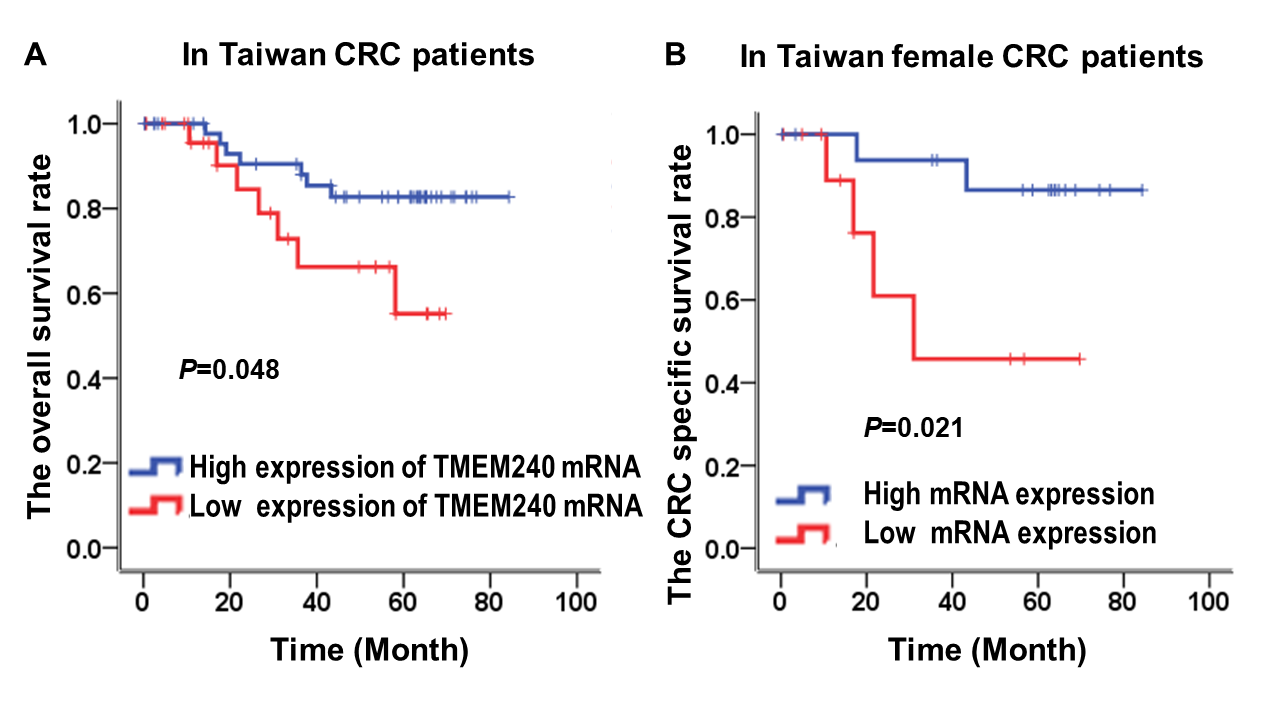
**

**Figure S5.** The Kaplan-Meier survival curves for (A) overall survival in Taiwanese colorectal carcinoma (CRC) patients and (B) cancer-specific survival in Taiwanese females are shown. *TMEM240* was considered to have low expression when the expression level in CRC tumors was 5-fold lower than that in normal tissues.

**Table S1. *TMEM240* mRNA expression and promoter hypermethylation in relation to the clinical parameters of the TCGA CRC dataset.^a^**

| **Characteristics** | | **Total n** | ***TMEM240* mRNA^b^**  **Low n (%) High n (%)** | | | | | **Total n** | ***TMEM240* Methylation^c^**  **Low n (%) High n ( %)** | | | | | | |  |
| --- | --- | --- | --- | --- | --- | --- | --- | --- | --- | --- | --- | --- | --- | --- | --- | --- |
|  |  |  |  |  |  | | |  |  | | |  | | | |  |
| **Overall** | | 456 | 389 | (85.3) | | 67 | (14.7) | 293 | 48 | (16.4) | 245 | | (83.6) | |  |  |
| **Age** | |  |  |  |  | |  |  |  |  | |  | |  | |  |
|  | < 65 | 184 | **150** | **(81.5)** | **34** | | **(81.5)^0.090^** | 136 | **31** | **(22.8)** | | **105** | | **(77.2)^0.008^** | |  |
|  | > 65 | 261 | **228** | **(87.4)** | **33** | | **(12.6)** | 146 | **16** | **(11.0)** | | **130** | | **(89.0)** | |  |
| **Race** | |  |  |  |  | |  |  |  |  | |  | |  | |  |
|  | Asian | 11 | **11** | **(100.0)** | **0** | | **(0.0)^0.265^** | 11 | **2** | **(18.2)** | | **9** | | **(81.8)^0.970^** | |  |
|  | Black or African American | 52 | **46** | **(88.5)** | **6** | | **(11.5)** | 51 | **8** | **(15.7)** | | **43** | | **(84.3)** | |  |
|  | White | 211 | **177** | **(83.9)** | **34** | | **(16.1)** | 201 | **34** | **(16.9)** | | **167** | | **(83.1)** | |  |
| **Sex** | |  |  |  |  | |  |  |  |  | |  | |  | |  |
|  | Male | 211 | **176** | **(83.4)** | **35** | | **(16.6)^0.391^** | 130 | **25** | **(19.2)** | | **105** | | **(80.8)^0.285^** | |  |
|  | Female | 234 | **202** | **(86.3)** | **32** | | **(13.7)** | 152 | **22** | **(14.5)** | | **130** | | **(85.5)** | |  |
| **Tumor Type** | |  |  |  |  | |  |  |  |  | |  | |  | |  |
|  | Adenocarcinoma | 383 | **330** | **(86.2)** | **53** | | **(13.8)^0.113^** | 245 | **38** | **(15.5)** | | **207** | | **(84.5)^0.180^** | |  |
|  | Mucinous | 60 | **47** | **(78.3)** | **13** | | **(21.7)** | 37 | **9** | **(24.3)** | | **28** | | **(75.5)** | |  |
| **Tumor Stage** | |  |  |  |  | |  |  |  |  | |  | |  | |  |
|  | I | 75 | **67** | **(89.3)** | **8** | | **(10.7)^0.323^** | 43 | **2** | **(4.7)** | | **41** | | **(95.3)^0.127^** | |  |
|  | II | 176 | **153** | **(86.9)** | **23** | | **(13.1)** | 112 | **22** | **(19.6)** | | **90** | | **(80.4)** | |  |
|  | III | 122 | **99** | **(81.1)** | **23** | | **(18.9)** | 79 | **14** | **(17.7)** | | **65** | | **(82.3)** | |  |
|  | IV | 61 | **50** | **(82.0)** | **11** | | **(18.0)** | 38 | **8** | **(21.1)** | | **30** | | **(78.9)** | |  |
| **Tumor Size** | |  |  |  |  | |  |  |  |  | |  | |  | |  |
|  | T0-T1 | 12 | **11** | **(91.7)** | **1** | | **(8.3)^0.509^** | 8 | **0** | **(11.8)** | | **8** | | **(100.0)^0.199^** | |  |
|  | T2-T4 | 433 | **367** | **(84.8)** | **66** | | **(15.2)** | 274 | **47** | **(17.2)** | | **227** | | **(82.8)** | |  |
| **Regional lymph nodes metastasis** | | | |  |  | |  |  |  |  | |  | |  | |  |
|  | N=0 | 266 | **234** | **(88.0)** | **32** | | **(12.0)^0.028^** | 166 | **26** | **(15.7)** | | **140** | | **(84.3)^0.566^** | |  |
|  | N>1 | 178 | **143** | **(80.3)** | **35** | | **(19.7)** | 115 | **21** | **(18.3)** | | **94** | | **(81.7)** | |  |
| **Distant metastasis** | | | |  |  | |  |  |  |  | |  | |  | |  |
|  | M=0 | 324 | **277** | **(85.5)** | **47** | | **(14.5)^0.359^** | 186 | **30** | **(16.1)** | | **156** | | **(83.9)^0.553^** | |  |
|  | M>1 | 63 | **51** | **(81.0)** | **12** | | **(19.0)** | 40 | **8** | **(20.0)** | | **32** | | **(88.0)** | |  |
| **MSI** | |  |  |  |  | |  |  |  |  | |  | |  | |  |
|  | No | 80 | **68** | **(85.0)** | **12** | | **(15.0)^0.168^** | 76 | **15** | **(19.7)** | | **61** | | **(80.3)^0.105^** | |  |
|  | Yes | 11 | **11** | **(100.0)** | **0** | | **(00.0)** | 11 | **0** | **(0.0)** | | **11** | | **(100.0)** | |  |
| **Kras mutation** | |  |  |  |  | |  |  |  |  | |  | |  | |  |
|  | No | 361 | **304** | **(84.2)** | **57** | | **(15.8)^0.355^** | 200 | **28** | **(14.0)** | | **172** | | **(86.0)^0.038^** | |  |
|  | Yes | 47 | **42** | **(89.4)** | **5** | | **(10.6)** | 45 | **12** | **(26.7)** | | **33** | | **(73.3)** | |  |
|  | |  |  |  |  | |  |  |  |  | |  | |  | |  |
|  |  |  |  |  |  | |  |  |  |  | |  | |  | |  |
|  | 1. These results were analyzed by Pearson's *X^2^* test. Significant *p* values are indicated by superscripts. For some categories, the number of samples (*n*) was lower than the overall number analyzed because clinical data were unavailable for these samples. 2. When the *TMEM240* expression level determined by RNA sequencing analysis of CRC tumors from the TCGA dataset was less than the median *TMEM240* expression level in adjacent normal colon tissues, a sample was considered to have low expression. 3. When the *TMEM240* methylation level (ΔAvg_β), as determined with the Illumina Infinium HumanMethylation450 BeadChip array, in CRC tumors from the TCGA dataset was higher than 0.25, a sample was considered to have hypermethylation. | | | | | | | | | | | | | | | |

**Table S2 List of primers sequences and their reaction conditions used in the present study**

| **Gene** | **primer** | **5’→3’sequences** | **Application** | **Size**  **(bp)** | **Tm (**$\mathbf{℃}$**)** |
| --- | --- | --- | --- | --- | --- |
| *GAPDH* | Forward  Reverse  Probe | AGCCACATCGCTCAGACAC  GCCCAATACGACCAAATCC  Roche Universal Probe # 60 | RT-PCR  Real-time | 66 | 60 |
| *TMEM240* | Forward  Reverse  Probe | ATCGCGTGCTTGATGGAC  GGATCACGTAGTGGATATGGTG  Roche Universal Probe # 88 | RT-PCR  Real-time | 125 | 60 |
| *BACTIN* | Forward  Reverse | TGGTGATGGAGGAGGTTTAGTAAGT  AACCAATAAAACCTACTCCTCCCTTAA | MSP-M | 132 | 60 |
| *TMEM240* | Forward  Reverse  Probe | TTTTTCGTTTATTATTACGATCGAC  CAAAAAAACGCTAACCTCTACG  TTTAGAATTATGAAGATTATGGTGTTC | MSP-M | 177 | 60 |
